# Individual- and City-Level Socioeconomic Factors and Tooth Loss among Elderly People: A Cross-Level Multilevel Analysis

**DOI:** 10.3390/ijerph17072345

**Published:** 2020-03-30

**Authors:** Mario Vianna Vettore, Janete M. Rebelo Vieira, José F. F. Gomes, Nara M. O. Martins, Yan N. L. Freitas, Gabriela de A. Lamarca, Maria A. B. Rebelo

**Affiliations:** 1 School of Dentistry, Federal University of Minas Gerais, Belo Horizonte 31270-901, Brazil; gabilamarca@ufmg.br; 2 Academic Unit of Oral Health, Dentistry and Society, School of Clinical Dentistry, University of Sheffield, Sheffield S10 2TA, UK; 3 School of Dentistry, Federal University of Amazonas, Manaus 69000-000, Brazil; jmrvieira@ufam.edu.br (J.M.R.V.); gomesjf@hotmail.com (J.F.F.G.); nara.munik@hotmail.com (N.M.O.M.); yannogueira@ufam.edu.br (Y.N.L.F.); augusta@ufam.edu.br (M.A.B.R.)

**Keywords:** edentulous, tooth loss, multilevel analysis, social inequity, elderly people

## Abstract

This study aimed to test the association of contextual and individual socioeconomic status with tooth loss among Brazilian elderly people aged 65–74 years. Data from 5435 elderly participants from the Brazilian National Oral Health Survey (2010) were linked to city-level data for 27 state capitals and the Federal District. Tooth loss was clinically assessed according to the number of missing natural teeth. Contextual social variables included Human Development Index income (HDI-income) and HDI-education. Individual socioeconomic measures were monthly family income and years of schooling. Covariates included sex, skin colour, number of residents per room and number of goods. Multilevel Negative Binomial regression models were used to estimate rate ratios (RR) and 95% confidence intervals between contextual and individual variables and tooth loss. Contextual and individual income and education measures were consistently associated with tooth loss. Elderly people living in cities with low HDI-income and low HDI-education were respectively 21% and 33% more likely to present tooth loss. Cross-level interaction suggested that the relationship of lower income and lower schooling with tooth loss is different across levels of city-level income and city-level education inequality, respectively. Public policies aiming to reduce the income and education gaps and preventive dental interventions are imperative to tackle tooth loss among elderly people.

## 1. Introduction

Despite the global decline in the occurrence of severe tooth loss observed over the last decades, Latin American countries still experience high prevalence of tooth loss [1]. In addition, incidence rates of severe tooth loss differ significantly between regions and countries, suggesting that tooth loss remains as one of the main oral health problems in some developing countries [1]. Tooth loss also causes functional and social impairments that negatively impact an individual’s quality of life due to reduction of chewing ability and the limitations of social interaction [2].

Recent oral health surveys conducted in Brazil revealed an overall decline of dental caries in most age groups [3]. However, the prevalence of tooth loss among elderly people remained stable according to the last national surveys when around half of the population aged between 65 and 74 years old experienced edentulism [3,4]. In the last survey, the missing component accounted for 91.9% of the total decayed, missing and filled teeth index (DMFT) in this age group. Furthermore, 15.4% of the elderly people needed both lower and upper total dental prosthesis [3]. Therefore, the high prevalence of tooth loss among Brazilian elders is a public health concern that needs to be addressed.

Private and public dental services are available in Brazil where public dental services are offered to all age groups by the Brazilian national health system named Unified Health System (Sistema Único de Saúde – SUS). Although oral health care is free of charge in the national health system, the Brazilian population faces socioeconomic barriers to access dental services. This results in inequities in the utilization of dental services, which is particularly critical for specialized dental treatments such as provision of dental prosthesis [4].

Secondary analysis of nationwide oral health surveys in different countries reported the influence of poor individual socioeconomic characteristics, such as family income, occupational history and educational level, on tooth loss among elders and adults [5,6,7,8]. These findings are in accordance with a recent systematic that concluded that lower levels of education and poor income were meaningful factors associated with edentulism among elderly people [9].

The possible role of contextual and individual socioeconomic inequalities on tooth loss among adults has been explored. Overall, these studies reported that adults living in socially deprived regions and in areas with higher levels of income inequality had a greater likelihood of having fewer natural teeth than those living in better-off regions [10,11,12,13,14]. However, studies exploring whether contextual social inequalities may influence tooth loss among elderly people are scarce [15].

The objective of this study was to investigate the associations between city- and individual-level income and educational inequalities and tooth loss among elderly people in Brazil using the 2010 Brazilian National Oral Health Survey (*SB Brasil Project*) data. It was hypothesized that elderly people residing in more socially disadvantaged cities and those from lower socioeconomic backgrounds present higher tooth loss than their counterparts from more advantageous backgrounds. It was also conjectured that contextual social inequalities modifies the relationship between individual social indicators and tooth loss in this age group.

## 2. Materials and Methods

### 2.1. Study Population and Data Collection

This cross-sectional study combined secondary individual- and city-level data from the last Brazilian National Oral Health Survey (*SB Brasil Project*) conducted in 2010 [16] and data from the Brazilian agency of United Nations Development Program [17]. Of the total 37,519 participants, 7619 elders aged 65 to 74 years participated in the survey. The analyzed sample included 5435 elderly people from the 26 Brazilian state capitals and the Federal District once the sample was considered to be representative of oral health conditions for these geographical areas.

The sample of the survey was obtained using a stratified multistage cluster sampling method where the census tracts and households were the primary and secondary sampling units, respectively. Further information of the sampling process is available elsewhere [18]. Participants were recruited from their homes where individual interviews and dental examinations were carried out [16]. Dental examinations were conducted by a calibrated dentist under natural light with sterilized instruments following the protocol of oral health surveys proposed by the World Health Organization [19]. All examiners obtained a minimum Kappa coefficient accepted of 0.65 for decayed, missing and filled teeth index [16].

The present study received ethical clearance from the Brazilian National Council of Ethics in Research (no. 15498) and was conducted in accordance with the Declaration of Helsinki. Informed consent was obtained from all participants before dental examination and interviews.

### 2.2. Variables and Theoretical Model

Tooth loss was the outcome assessed as a count variable according to the number of missing natural teeth. The independent individual variables were age, gender, self-reported skin colour, monthly family income, years of schooling, number of residents per room, and number of goods in the household. Skin colour was self-reported according to the Brazilian Institute of Geography and Statistics classification (white, yellow, indigenous, brown and black). Monthly family income was recorded in Brazilian Reais (R$) (≤R$ 500, R$ 501–1500, R$ 1501–2500 and ≥R$ 2501). One Brazilian Real corresponded to 0.586 USD. Years of schooling was measured according to the number of years at school concluded without failure (0–4, 5–8, 9–11, ≥12). The number of residents per room was assessed by dividing the number of residents (excluding housemaids) by the number of bedrooms in the house.

The independent contextual variables used to assess income and educational social inequalities were Human Development Index income (HDI-income) and HDI-education [17]. The former reflects the living standards and purchasing power of the population and it is assessed according to the Municipal Gross Income per capita, while the latter is measured based on the average years studied of the population [17]. The distribution of HDI-income and HDI-education was categorized into a three-level ordinal variables (low, moderate and high) according to tertiles of their distribution in order to assess whether there was a gradient in the distribution of tooth loss in relation to the contextual social indicators.

**Figure 1:**
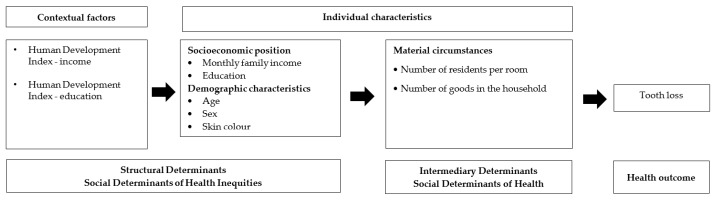
Theoretical model for the study of contextual and individual structural social determinants of tooth loss in elderly people.

The adapted version of the Conceptual Framework for Action on the Social Determinants of Health was used to organize the contextual and individual structural determinants of tooth loss into second (city) and first (individual) levels [20] (Figure 1).

### 2.3. Statistical Analysis

The mean of tooth loss and 95% confidence intervals (95% CI) were estimated according to the individual and contextual independent variables. The association of contextual and individual independent variables with tooth loss was assessed by multilevel Negative Binomial regression using fixed effects models with random intercept and logit function to estimate adjusted rate ratios (ARRs) and 95% CI.

Multilevel models were used to estimate the variation of tooth loss between cities (random effects) and the effects of contextual variables on tooth loss adjusted for individual sociodemographic characteristics (fixed effects). Initially, crude associations examined the association between independent variables and tooth loss. Independent variables that achieved a *p*-value < 0.10 were considered in the multivariable analysis.

The analysis was extended to test for cross-level interactions by including interaction terms for combinations of individual-level income and education and city-level income and education disadvantage on tooth loss. The substantive focus of the interaction analyses was on whether associations between individual income and education differed across cities that varied in their level of socioeconomic disadvantage.

The variance and standard error of tooth loss between cities (random effects) were used to evaluate the variation of outcomes at the contextual level. The interaction between contextual and individual social variables was evaluated by comparing measures of model adjustments (deviance) between statistical models. Deviance measures between the models with and without the interaction variable were compared using the Chi-square test. The difference between the deviances follows a Chi-square distribution with n-1 degrees of freedom, where n is the number of parameters included in the models.

IBM SPSS^®^ 24.0 (IBM Corp., Armonk, NY, USA) was used for descriptive analyses considering the complex sample and sample weights. Multilevel Negative Binomial regression analyses were conducted using STATA^®^ 16.0 (StataCorp, College Station, Texas, TX, USA).

## 3. Results

### 3.1. Tooth Loss

The mean of missing natural teeth among Brazilian elders was 23.9, ranging from 18.6 (CI 95% 16.0–21.2) to 26.9 (CI 95% 25.1–28.7) between the state capitals and the Federal District. The distribution of tooth loss according to the contextual and individual variables is presented in Table 1. Tooth loss was lower in cities with higher HDI-income and HDI-education.

### 3.2. Unadjusted Association between Contextual and Individual Variables and Tooth Loss

The null Negative Binomial multilevel model revealed significant difference among the 27 cities concerning the mean of tooth loss (*p* < 0.001). The distribution of tooth loss between cities remained significant for both contextual variables as city-level variance remained statistically significant when HDI-income and HDI-education measures were added to the null model (*p* < 0.001).

The crude multilevel negative binomial regression analysis presents the unadjusted crude RRs and 95% CIs for the number of missing natural teeth by contextual and individual variables (Table 2). There was a significant association between HDI-income and tooth loss. In addition, age, sex, skin colour, monthly family income, years of schooling, number of residents per room and number of goods in the household were significantly associated with tooth loss.

### 3.3. Association between HDI-Income and Individual Variables and Tooth Loss

Table 3 presents the multivariate multilevel negative binomial regression between contextual measure of income, individual demographic and socioeconomic variables and tooth loss. Higher age, elderly women, low monthly family income and lower years of schooling remained statistically associated with tooth loss. A lower HDI-income was statistically associated with tooth loss (ARR = 1.21, 95% CI 1.13–1.30). The models with and without the interaction variable monthly family income x HDI-income did differ statistically (Chi-square test = 19.10, degrees of freedom (df) = 6, *p* = 0.004), suggesting the cross-level interactions between HDI-income and monthly family income was significant.

### 3.4. Association between HDI-Education and Individual Variables and Tooth Loss

The adjusted associations between contextual measure of education, individual demographic and socioeconomic variables and tooth loss are shown in Table 4. Older participants, female elders, individuals with low monthly family income and lower years of schooling presented higher mean of tooth loss. Elderly people living in the cities with moderate (ARR = 1.32, 95% CI 1.18–1.47) and low HDI-education were more likely to have tooth loss (ARR = 1.33, 95% CI 1.22–1.46). Cross-level interaction was statistically significant between years of schooling x HDI-education once the models with and without the interaction term differed statistically (Chi-square test = 49.58, df = 6, *p* < 0.001). Elderly people with 0–4 years of schooling and living in cities with low HDI-education were 1.34 times more likely to have tooth loss than those living in cities with high HDI-education and 12 years of schooling or more.

The adjusted multilevel negative binomial regression on the association between monthly family income and tooth loss according to the tertiles of HDI-income is shown in Table 5. The relationship between low monthly family income and tooth loss was stronger in low HDI-income cities than in high HDI-income ones. The mean of tooth loss was 1.21 times higher in elders with monthly family income ≤R$ 500 than those with monthly family income ≥R$ 2501 among those living in high HDI-income. Elderly people with monthly family income ≤R$ 500 were 1.27 more likely to have higher tooth loss than those with monthly family income ≥R$ 2501 if they lived in low HDI-income cities.

Table 6 includes the adjusted multilevel negative binomial regression on the association between years of schooling and tooth loss according to the tertiles of HDI-education. The association between low schooling and tooth loss was stronger in low HDI-education cities than in high HDI-education ones. The mean of tooth loss was 1.35 times higher in elders with 0–4 years of schooling than those with 12 years of schooling among those living in high HDI-education cities. Elderly people with 0–4 years of schooling were 1.67 times more likely to have higher tooth loss than those with 12 years of schooling if they lived in low HDI-education cities.

## 4. Discussion

This study examined associations between city-level disadvantage, individual socioeconomic background and tooth loss among elderly people using a nationwide and representative sample of Brazilian elders aged 65–74 years of age. Overall, the multilevel analysis revealed the following findings. First, contextual social inequalities related to city-level income and education disparities were consistently associated with tooth loss after adjusting for individual level socioeconomic status. This result suggests that the socioeconomic characteristics of the city may have important implications for tooth loss. Second, there was a dose-response relationship between individual low socioeconomic status and tooth loss, suggesting a social gradient on tooth loss. Finally, significant cross-level interaction between individual- and community-level income and educational social inequalities on tooth loss were observed, which indicates a harmful effect of living in cities with low income and poor school achievement on tooth loss among those with low income and low schooling. Therefore, the present findings support the influence of contextual and individual social inequalities on oral health among elderly people in developing countries.

Our findings on the link between social inequalities and tooth loss are consistent with previous population-based single-level studies conducted in the Uruguay, Korea and Japan despite the methodological differences in the sample size, sample age group, sampling procedures, definition of outcome (e.g., severe tooth loss, number of remaining teeth) and measures of socioeconomic status (e.g., occupational history, income) [6,7,8]. Overall, these studies showed a high prevalence of tooth loss in the studied samples. Furthermore, lower individual socioeconomic status was a meaningful factor of tooth loss among older adults and elderly people after adjusting for possible confounders [6,7,8]. Only one multilevel study reported significant associations of contextual social disadvantage and individual socioeconomic position with complete denture need [15]. There are methodological differences between our study and da Veiga et al. [15], including the difference in the definition of the outcome, the contextual measures of social inequalities and the analytical approach.

Other research on social inequalities of tooth loss has used different methodological approaches to assess contextual socioeconomic conditions. Some studies have used a single measure of income inequality (e.g., Gini Index) [10,13], while another assessed the relationship between multiple contextual measures and tooth loss at the same time [12,14,15]. Due to possible similarities between the different area-level socioeconomic status measures, one study used component analysis to extract a single factor in order to aggregate different measures of socioeconomic status avoiding multicolinearity [11]. The relationship between HDI, tooth loss and completed denture need has been shown [12,14,15]. Chalub et al. observed that the prevalence rates of four definitions of functional dentition were greater among adults living in cities with very high HDI (≥ 0.800) than those from very low, low and medium (≤ 0.699) HDI [12]. Similarly, elderly people living in cities with low HDI, according to the median of HDI, were more likely to present higher needs of complete denture [15]. The present research tested the association of the component income and education of HDI with tooth loss. Although HDI components are part of the same index and therefore these measures are correlated [17], they were considered separate indicators of contextual social inequalities in this study since they assess different social characteristics. This allowed us to examine whether one or both contextual measures were important for tooth loss among elderly people. Nonetheless, the association of HDI-income and HDI-education with tooth loss was assessed in separate multivariate multilevel analyses to prevent overadjustment bias.

As far as the authors are aware, this is the first study investigating cross-level interactions between contextual social inequality, individual-level socioeconomic status and tooth loss. Our results showed that the association of monthly family income and years of schooling with tooth loss differed across cities with different levels of HDI-income and HDI-education. Older people from low-income families living in cities with the low-wage population showed greater tooth loss. Similarly, elders with lower levels of education living in cities where the population presents worst education attainment presented the highest mean of tooth loss. This phenomenon has been described as double disadvantage in the scientific literature. Double disadvantage has also been reported in previous studies on oral epidemiology [21,22]. Oral health disparities related to racial/ethnic background among children were greater in wealthy schools than in deprived schools [21]. Differences in dental care utilization between older adults with different levels of education were attenuated by country-specific dentist density [22].

Some limitations of this study must be considered. First, the studied sample included elderly people aged from 65 to 74 years and as thus the implications of our findings to other age groups should be cautious. Second, this study used cross-sectional data and causal inferences are not allowed. Third, historical and cultural differences on the experience and meaning of tooth loss and denture wearing have been highlighted [23]. Thus, our findings might not apply to countries with different cultural characteristics. Fourth, the second-level socioeconomic characteristics referred to the state capitals and the Federal District in Brazil, which are large cities with marked social inequalities across their neighbourhoods that were not considered in this study. Fifth, possible predictors of tooth loss, including health-related behaviours (e.g., smoking, alcohol consumption) and other individual socioeconomic factors (e.g., occupation) among elderly people were not assessed. It is important to highlight that the aforementioned predictors are also related to income and education level. Finally, although fluoride water has been identified as an important protective factor of tooth loss among adults [12], water fluoridation was not assessed. In Brazil, fluoridation of tap water came into force in the 1970s and thus the participants of the 2010 Brazilian National Oral Health Survey did not benefit from the effects of fluoridate water [5].

## 5. Conclusions

Individual and contextual measures of income and education were significantly associated with tooth loss in elderly people in Brazil. The remarkable social inequalities in the country suggests the need of effective public policies to reduce the income and education gaps across difference socioeconomic groups in order to tackle tooth loss.

## Figures and Tables

**Table 1:** Mean of number of missing natural teeth according to contextual factors and individual characteristics among elderly people.

Variables	Number of Missing Natural Teeth	
	Mean ^†^	95%CI ^‡^
Contextual variables		
HDI-Income		
Low	24.3	23.7–24.9
Moderate	25.0	23.6–26.4
High	22.4	21.4–23.4
HDI-Education		
Low	24.0	23.2–24.8
Moderate	24.4	23.2–25.5
High	21.9	20.8–23.1
Individual variables		
Sex		
Male	22.9	21.8–24.0
Female	24.5	23.6–25.4
Skin colour		
White	23.2	22.0–24.4
Yellow	23.9	18.3–29.6
Indigenous	20.2	15.4–25.1
Brown	24.8	24.0–25.6
Black	24.8	23.3–26.4
Monthly family income (R$) ^§^		
> 2500	18.2	16.4–20.1
1501–2500	22.9	21.7–24.1
501–1500	25.1	24.4–25.7
≤ 500	27.3	26.1–28.5
Years of schooling		
0–4 years	25.7	25.1–26.4
5–8 years	24.2	22.7–25.7
9–11 years	22.4	19.3–25.4
≥ 12 years	16.1	14.0–18.1

† Mean were estimated by complex samples; ‡ 95% confidence interval; § One Brazilian Real corresponded to 0.586 US dollars when the study was conducted.

**Table 2:** Crude associations of contextual factors and individual characteristics with tooth loss among elderly people determined by multilevel negative binomial regression.

Variable	Variance	RR ^†^	95%CI ^‡^
Null model	0.011 (0.005) *		
Level 2: Contextual variables			
HDI – Income (ref. = High)	0.070 (0.001) *		
Moderate		1.11	1.04–1.20
Low		1.14	1.06–1.21
HDI – Education (ref. = High)	0.081 (0.012) *		
Moderate		1.06	0.98–1.16
Low		1.08	1.01–1.16
Level 1: Individual variables			
Age		1.02	1.01–1.02
Sex (ref. = Male)			
Female		1.09	1.06–1.12
Skin colour (ref. = White)			
Yellow		1.03	0.91–1.17
Indigenous		1.00	0.86–1.16
Brown		1.08	1.05–1.11
Black		1.04	0.99–1.09
Monthly family income (R$) (Ref. ≥ 2500) ^§^			
1501–2500		1.31	1.25–1.37
501–1500		1.38	1.33–1.44
≤ 500		1.42	1.35–1.50
Years of schooling (ref. ≥ 12)			
9–11 years		1.32	1.25–1.40
5–8 years		1.51	1.44–1.59
0–4 years		1.62	1.55–1.70
Number of residents per room		1.03	1.01–1.05
Number of goods in the household		0.97	0.96–0.97

* *p* < 0.01; † Rate Ratio; ‡ 95% confidence interval; § One Brazilian Real corresponded to 0.586 US dollars when the study was conducted.

**Table 3:** Adjusted associations of HDI-Income (contextual factor) and individual characteristics with tooth loss among elderly people determined by multilevel negative binomial regression.

Variables	ARR *	95%CI †
Individual variables		
Age	1.02	1.01–1.02
Sex (ref. = Male)		
Female	1.08	1.05–1.11
Skin/colour (ref.-White)		
Yellow	0.96	0.85–1.08
Indigenous	0.93	0.81–1.06
Brown	1.01	0.98–1.04
Black	0.96	0.92–1.00
Monthly family income (ref. ≥ 2500) ‡		
1501–2500	1.25	1.16–1.34
501–1500	1.29	1.21–1.37
≤ 500	1.41	1.25–1.60
Years of schooling (ref. ≥ 12)		
9–11	1.25	1.18–1.31
5–8	1.38	1.31–1.45
0–4	1.45	1.38–1.52
Number of residents per room	1.01	0.99–1.03
Number of goods in the household	0.99	0.99–1.00
Contextual variables	1.01	0.99–1.03
HDI – Income (ref. = High)		
Moderate	1.09	1.00–1.18
Low	1.21	1.13–1.30
Cross-level interaction term (ref. = High HDI-Income x Monthly family income ≥ 2500)		
HDI-Income x Monthly family income †		
Moderate HDI-Income x Monthly family income 1501–2500	1.07	0.96–1.19
Moderate HDI-Income x Monthly family income 501–1500	1.11	1.01–1.22
Moderate HDI-Income x Monthly family income ≤ 500	1.15	1.02–1.31
Low HDI-Income x Monthly family income 1501–2500	1.11	1.01–1.22
Low HDI-Income x Monthly family income 501–1500	1.17	1.08–1.27
Low HDI -Income x Monthly family income ≤ 500	1.28	1.11–1.47

* Adjusted Rate Ratio; † 95% confidence interval; ‡ One Brazilian Real corresponded to 0.586 US dollars when the study was conducted.

**Table 4:** Adjusted associations of HDI-Education (contextual factor), individual characteristics with tooth loss among elderly people determined by multilevel negative binomial regression.

Variables	ARR *	95%CI †
Individual variables		
Age	1.02	1.01–1.02
Sex (ref. = Male)		
Female	1.08	1.05–1.11
Skin/colour (ref. = White)		
Yellow	0.96	0.86–1.08
Indigenous	0.93	0.81–1.06
Brown	1.01	0.98–1.04
Black	0.96	0.92–1.01
Monthly family income (ref. > 2500) ‡		
1501-2500	1.18	1.13–1.24
501-1500	1.19	1.14–1.25
≤ 500	1.23	1.16–1.30
Years of schooling (≥ 12)		
9–11	1.37	1.25–1.50
5–8	1.69	1.56–1.84
0–4	1.76	1.81–1.90
Number of residents per room	1.01	0.99–1.02
Number of goods in the household	0.99	0.98–0.99
Contextual variables		
HDI – Education (ref. = High)		
Moderate	1.32	1.18–1.47
Low	1.33	1.22–1.46
Cross-level interaction term		
HDI-Education x years of schooling (ref. = High HDI-Education x years of schooling ≥ 12)		
Moderate HDI-Education x years of schooling 9–11	0.97	0.84–1.11
Moderate HDI-Education x years of schooling 5–8	0.96	0.85–1.09
Moderate HDI-Income x monthly family income 0–4	1.00	0.89–1.12
Low HDI-Education x years of schooling 9–11	1.18	1.01–1.29
Low HDI-Education x years of schooling 5–8	1.33	1.19–1.49
Low HDI-Education x years of schooling 0–4	1.34	1.21–1.48

*Adjusted Rate Ratio; † 95% confidence interval; ‡ One Brazilian Real corresponded to 0.586 US dollars when the study was conducted.

**Table 5:** Association between monthly family income and tooth loss among elderly people according to Human of Development Index income determined by multilevel negative binomial regression.

Variables	High HDI-Income *N* = 2287		Moderate HDI- Income *N* = 1530		Low HDI- Income *N* = 1618	
	ARR *	95%CI †	ARR *	95%CI †	ARR *	95%CI †
Monthly family income (ref. > 2500) ‡						
1501-2500	1.19	1.12–1.26	1.18	1.08–1.29	1.20	1.11–1.31
501-1500	1.19	1.13–1.27	1.15	1.05–1.25	1.22	1.13–1.33
≤ 500	1.21	1.12–1.30	1.23	1.04–1.45	1.27	1.14–1.42

* Adjusted Rate Ratio. Estimates are adjusted for age, sex, years of schooling and number of goods; † 95% confidence interval; ‡ One Brazilian Real corresponded to 0.586 US dollars when the study was conducted.

**Table 6:** Association between years of schooling and tooth loss among elderly people according to Human of Development Index education determined by multilevel negative binomial regression.

Variables	High HDI-Education *N* = 2187		Moderate HDI-Education *N* = 1616		Low HDI-Education *N* = 1598	
	ARR *	95%CI †	ARR *	95%CI †	ARR *	95%CI †
Years of schooling (ref. ≥ 12)						
9–11	1.22	1.13–1.32	1.18	1.06–1.32	1.34	1.21–1.49
5–8	1.30	1.21–1.40	1.25	1.14–1.39	1.64	1.48–1.81
0–4	1.35	1.26–1.45	1.37	1.24–1.50	1.67	1.51–1.83

* Adjusted Rate Ratio. Estimates are adjusted for age, sex, monthly family income and number of goods.; † 95% confidence interval
